# Impact of intra‐tumoral immunity on predicting response and survival after neoadjuvant cisplatin‐based chemotherapy in patients with muscle invasive bladder cancer

**DOI:** 10.1002/cam4.70088

**Published:** 2024-08-09

**Authors:** Alberto Mendoza‐Valderrey, Jane Choe, Daria M. Kessler, Gianna Jimenez, Xinmin Li, Steven Kolker, Warren Allen, Jennifer A. Linehan, Przemyslaw W. Twardowski, Maria Libera Ascierto

**Affiliations:** ^1^ Translational Cancer Immunology and Immunotherapy Department Saint John's Cancer Institute, Providence Saint John's Health Center Santa Monica California USA; ^2^ Rosalie and Harold Rae Brown Cancer Immunotherapy Research Program Saint John's Cancer Institute, Providence Saint John's Health Center Santa Monica California USA; ^3^ Department of Urologic Oncology Saint John's Cancer Institute, Providence Saint John's Health Center Santa Monica California USA; ^4^ Bioinformatic Department Saint John's Cancer Institute, Providence Saint John's Health Center Santa Monica California USA; ^5^ Technology Center for Genomics and Bioinformatics, UCLA Los Angeles California USA; ^6^ Pathology Department Providence Saint John's Health Center Santa Monica California USA; ^7^ Translational Cancer Immunology University of Glasgow Glasgow UK

**Keywords:** biomarkers, bladder cancer, digital pathology, neoadjuvant

## Abstract

**Background:**

Neoadjuvant cisplatin‐based chemotherapy (NAC) followed by cystectomy is the standard of care for patients with muscle‐invasive bladder cancer (MIBC). Pathologic complete response (pCR) is associated with favorable outcomes, but only 30%–40% of patients achieve that response. The aim of this study is to investigate the role played by the Tumor and Immune Microenvironment (TIME) in association with the clinical outcome of patients with MIBC undergoing NAC.

**Methods:**

Nineteen patients received NAC and were classified as pCR (*n* = 10) or non‐pCR (*n* = 9). Bulk RNA‐seq and immune protein evaluations using Digital Spatial Profiling (DSP) were performed on formalin‐fixed paraffin‐embedded (FFPE) tumor biopsies collected before NAC (baseline). Immunohistochemistry (IHC) evaluation focused on CD3 and CD20 expression was performed on baseline and end‐of‐treatment (EOT) FFPEs. Baseline peripheral blood was assessed for lymphocyte and neutrophil counts. Kaplan–Meier analyses and Cox PH regression models were used for survival analyses (OS).

**Results:**

In the periphery, pCR patients showed lower neutrophil counts, and neutrophil/ lymphocyte ratio (NLR) when compared to non‐pCR patients. In the tumor microenvironment (TME), gene expression analysis and protein evaluations highlighted an abundance of B cells and CD3^+^ T cells in pCR versus non‐pCR patients. On the contrary, increased protein expression of ARG1^+^ cells, and cells expressing immune checkpoints such as LAG3, ICOS, and STING were observed in the TME of patients with non‐pCR.

**Conclusions:**

In the current study, we demonstrated that lower NLR levels and increased CD3^+^ T cells and B cell infiltration are associated with improved response and long‐term outcomes in patients with MIBC receiving NAC. These findings suggest that the impact of immune environment should be considered in determining the clinical outcome of MIBC patients treated with NAC.

## 1 INTRODUCTION

Bladder cancer is the fourth and eighth most common cancer in men and women, respectively, in Western countries. Urothelial carcinoma (UC) is the most frequent histologic type of bladder cancer and is known for its immunogenicity. Approximately 30% of patients present with muscle‐invasive bladder cancer (MIBC) at first diagnosis. MIBC is a chemotherapy‐sensitive disease and cisplatin‐based neoadjuvant chemotherapy (NAC) administered before local definitive therapy (either cystectomy or bladder‐preserving chemoradiation in selected patients) represents the standard of care.^1^ Cisplatin eligibility criteria are defined by the Galsky criteria, which include creatinine clearance ≥60 mL/min, adequate heart and hearing function, good performance status, and lack of significant neuropathy. About 50% of patients with MIBC are considered ineligible for cisplatin chemotherapy because of medical comorbidities, particularly suboptimal renal function. Although NAC can induce pathological responses and statistically improve survival, only 30%–40% of patients experience a major response, defined as the absence of muscle‐invasive disease and lack of lymph node metastasis at the time of cystectomy.^2^ Nonresponding patients are unlikely to derive clinical benefit and are exposed to substantial toxicity and they experience a delay in definitive local therapy.^3^ Therefore, there is an urgent need to develop clinically useful predictive biomarkers to refine patient selection involving NAC for more precise delivery of care. The anti‐tumor mechanism of cisplatin is based on DNA damage through the formation of adducts that interfere with DNA replication and transcription. For this reason, numerous studies have analyzed the role of alterations in DNA repair genes as potential predictive biomarkers of response to cisplatin. In a study conducted in 2015, mutations in ATM serine/threonine kinase (*ATM*), RB transcriptional corepressor 1 (*RB1*) and FA complementation group C (*FANCC*) have been observed to correlate with pathological response and survival in MIBC patients treated with a combination of methotrexate, vinblastine, adriamycin, and cisplatin (MVAC).^4^ These results were confirmed in a validation cohort of patients treated with Gemcitabine + Cisplatin.^5^ Additionally, *ERCC2* mutations, identified in 12% of urothelial carcinomas, were linked to loss of the repair capacity of the nucleotide excision repair (NER) pathway and with sensitivity to platinum.^6^ Another study involving 50 MIBC patients treated with NAC revealed *ERCC2* mutations in 36% of patients who achieved pCR, while no mutations were found in patients with residual tumors. These results were corroborated in a validation series in which *ERCC2* mutations were detected in 8/20 (40%) of responders compared to 2/28 (7%) of non‐responders.^7^ Despite the potential of *ERCC2* mutations as predictive biomarkers for NAC response, their clinical application is limited due to their low frequency, as 60% of patients with a pCR did not exhibit these mutations. The molecular characterization of MIBC conducted by Robertson in 2017^8^ also provided a framework to guide the selection of optimal therapy thus further highlighting its importance on the patient's survival after receiving NAC therapy. However, the discordant findings regarding using MIBC molecular subtyping in treatment decisions^9^, ^10^, ^11^, ^12^ suggest that consideration of additional and simpler patient selection strategies is required. It is worth noting that cisplatin‐based chemotherapy has been associated with favorable immune‐modulatory effects in model systems,^13^, ^14^ suggesting a key role played by lymphocytic infiltrate in chemotherapy‐based regimens administered in adjuvant and neoadjuvant setting.

In the present study, we investigated on a retrospective cohort of 19 patients with MIBC undergoing NAC and with available tumor specimens and systemic immune correlates available prior to treatment initiation (baseline) and end of treatment (EOT), the role played by peripheral immunogenicity and Tumor and Immune Microenvironment (TIME) in patients undergoing NAC. In doing so, a particular focus on the role played by systemic lymphocytes and neutrophils counts and tumor‐infiltrating B cells and CD3^+^ T cells on NAC response has been here adopted.

## 2 MATERIALS AND METHODS

The clinical, molecular, and immunologic data from 19 patients with MIBC treated with neoadjuvant cisplatin‐based chemotherapy at the Saint John's Health Center (Santa Monica, CA) between 2016 and 2022 were evaluated in this study, which was approved by Providence Health Center Institutional Review Board (IRB). Patients consented to the collection of blood and/or tumor specimens for research. Baseline cisplatin‐based NAC and end of treatment (EOT)/surgery time‐points were evaluated. Detailed information for the patient cohort evaluated in this study is shown in Table 1.

**TABLE 1:** Clinical information of MIBC patients treated with neoadjuvant cisplatin‐based chemotherapy.

Patient #	Baseline FFPE Sample ID	Post‐treatment FFPE Sample ID	Neoadjuvant Tx (Date of Tx)	Response^b^	Date of surgery	OS (months)^c^	Patient gender	Patient age at time of Tx initiation
1^a^	BCNA1‐A^d^, ^e^, ^f^	BCNA1‐B^f^	Cis + Gem ‐ (02/2016)	Non‐pCR	5/18/2016	90	M	77
2^a^	BCNA2‐A^d^, ^f^	NA	Cis + Gem ‐ (05/2016)	Non‐pCR	8/29/2016	87	M	75
3	BCNA3‐A^d^, ^f^	BCNA2‐B^f^	MVAC ‐ (08/2016)	pCR	11/21/2016	84	M	60
4^a^	BCNA4‐A^d^, ^f^	NA	Cis + Gem ‐ (07/2017)	pCR	1/22/2018	73	M	77
5^a^	BCNA5‐A^d^, ^e^, ^f^	BCNA4‐B^f^	Cis + Gem ‐ (10/2017)	pCR	3/5/2018	70	M	62
6^a^	BCNA7‐A^d^, ^e^, ^f^	NA	Cis + Gem ‐ (01/2018)	pCR	5/25/2018	67	M	72
7	BCNA10‐A^d^, ^f^	BCNA9‐B^f^	MVAC ‐ (10/2018)	Non‐pCR	3/22/2019	9	M	74
8^a^	BCNA11‐A^d^, ^e^, ^f^	BCNA10‐B^f^	Cis + Gem ‐ (01/2019)	Non‐pCR	3/25/2019	36	M	78
9^a^	BCNA12‐A^d^, ^e^, ^f^	BCNA11‐B^f^	Cis + Gem ‐ (03/2019)	Non‐pCR	6/21/2019	4	M	58
10^a^	BCNA13‐A^d^, ^f^	NA	Cis + Gem ‐ (06/2020)	pCR	9/25/2020	5	M	82
11^a^	BCNA15‐A^d^, ^e^, ^f^	BCNA14‐B^f^	Cis + Gem ‐ (11/2020)	pCR	2/24/2021	33	M	57
12^a^	BCNA17‐A^d^, ^f^	NA	Cis + Gem + Pembro ‐ (09/2018)	pCR	NA	43	F	73
13^a^	BCNA18‐A^d^, ^f^	NA	MVAC ‐ (2/4/22)	pCR	3/18/2022	18	M	53
14^a^	NA	NA	Cis + Gem ‐ (03/2016)	Non‐pCR	5/16/2016	89	M	71
15^a^	NA	NA	Cis ‐ (11/2020)	Non‐pCR	1/19/2021	33	M	83
16^a^	NA	NA	Cis + radiation ‐ (9/2018)	pCR	NA	59	F	78
17^a^	NA	NA	Cis + Gem ‐ (1/2022)	Non‐pCR	4/29/2022	19	F	76
18^a^	NA	NA	Carbo + Cis + Gem ‐ (2/2022)	Non‐pCR	NA	2	M	79
19^a^	NA	NA	Cis + Gem ‐ (7/2022)	pCR	10/14/2022	13	M	61

*Note*: List of specimens derived from patients with MIBC evaluated in the study.

Abbreviations: Ci, cisplatin; Gem, gentamicin; MVA, methotrexate vinblastine sulfate doxorubicin hydrochloride cisplatin; NA, not applicable; OS, overall survival; Tx, treatment.

^a^ Had available lymphocytes and neutrophils counts assessed in the peripheral blood at the time of treatment initiation.

^b^ Clinical response to neoadjuvant cisplatin‐based chemotherapy assessed by pathological evaluation.

^c^ Overall survival (OS) from the end of treatment calculated in months.

^d^ Tumor specimens assessed by whole gene expression profile conducted on selected tumor area on FFPEs.

^e^ Tumor specimens assessed by Digital Spatial Profile using GeoMx NanoString.

^f^ FFPEs assessed by IHC.

A total number of 20 serial 5‐μm‐thick sections from formalin‐fixed, paraffin‐embedded (FFPE) specimens were obtained from a total of 13 MIBC patients. Baseline cisplatin‐based NAC FFPE specimens were available from 13 MIBC patients (8 pCR, 5 non‐pCR) and among them, 7 patients (3 pCR, 4 non‐pCR) also had matched FFPE specimens at EOT/surgery timepoint.

Furthermore, a total of 17 peripheral blood samples collected at baseline cisplatin‐based NAC (9 pCR, 8 non‐pCR) were also available for evaluation of lymphocyte and neutrophil counts.

### 2.1 Clinical response assessment

Clinical response, defined as pCR, refers to the absence of muscle‐invasive disease and lack of lymph node metastasis at the time of cystectomy.^2^ In case cystectomy was not performed, pCR was defined as the absence of muscle invasive disease observed in post‐treatment targeted and systematic cystoscopy biopsies. Non clinical response, defined as non‐pCR, referred to the presence of muscle invasive disease and lymph node metastasis at the time of cystectomy.

### 2.2 mRNA gene expression profiling and analysis

Tumoral regions were carefully identified by the pathologist and were manually dissected from 5‐μm tissue Formalin‐fixed, paraffin‐embedded (FFPE) sections as previously described.^15^ Total RNA was isolated from selected tumor areas with the High Pure RNA Paraffin Kit (Roche Diagnostics GmbH, Mannheim, Germany) following the manufacturer's guidelines. The quality and integrity of extracted RNA was assessed using the Eukaryote Total RNA Pico Kit on the Agilent 2100 Bioanalyzer (Agilent Technologies, Santa Clara, CA). The extracted total RNA was subjected to RNAseq and further analyzed in a total of 13 FFPE specimens derived from baseline tumors of pCR and non‐pCR patients. Briefly, ribosomal RNA (rRNA) was depleted and RNA‐seq libraries were prepared using KAPA Stranded RNA‐Seq Kit with RiboErase Kit (Roche). Single‐indexed libraries were pooled and sequenced on the Illumina NovaSeq 6000 platform for the PE 2 × 100 run. A data quality check was done on Illumina SAV. Demultiplexing was performed with Illumina Bcl2fastq v2.19.1.403 software. Initial analysis included alignment with TopHat2,^16^ and gene expression counts were obtained using cufflinks. FastQC was used for quality control. Data were processed in the R programming environment (v. 4.3.0)^17^ using the EdgeR^18^ package with a negative binomial model and common dispersion estimates to calculate the Differentially Expressed Genes (DEG) between the groups of interest. A *p* value ≤0.01 was considered statistically significant. Functional analysis was performed using the Ingenuity Pathway Analysis (IPA) software.

### 2.3 In silico data analysis

Data set from an independent cohort,^19^ containing RNA‐seq data with gene expression quantification including 93 MIBC patients, available from the Gene Expression Omnibus (GEO) database GSE31684 were used to conduct external validation. Patients receiving chemotherapy (*n* = 35) were removed from the initial cohort, so the final cohort comprised 58 patients for further bioinformatics and survival analysis.

### 2.4 Survival analysis

Overall survival considered as the clinical endpoint was calculated from EOT to the date of death or last follow‐up. Survival analyses were conducted according to lymphocyte counts, neutrophil counts, NLR, and mRNA expression data for *CD3*G or *CD19*. In all cases, median expression levels were used as cut off. For analysis conducted on RNA‐seq expression data, the median values of mRNA expression were calculated using R software (v. 4.3.0)^17^ through the GEOquery^20^ package (v2.70.0). Survival analyses were performed using the Kaplan–Meier non‐parametric method and log‐rank test. Survival curves were plotted using Stats Kingdom (https://www.statskingdom.com/kaplan‐meier.html). *p* values were calculated using the chi‐squared statistic using effect size of 0.3.

### 2.5 Digital spatial profiling (DSP)

High‐plex proteomic analyses with spatial resolution were conducted using GeoMx DSP (NanoString Technologies, Seattle, WA, USA) following instructions elsewhere described.^21^ FFPE sections were deparaffinized and incubated with a mixture of detection and morphological markers.

In detail, *n* = 20 Areas of Illumination (AOIs) derived from 3 available FFPE specimens from baseline pCR and *n* = 16 AOIs derived from 3 available FFPE specimens from non‐pCR were analyzed using DSP. Morphological markers ‐used to visualize tissue compartments and regions of interest (ROIs), included Syto13 for nucleus, pan‐cytokeratin (PanCk) for tumors, and CD45 for immune cells. The detection of 59 antibodies including one core panel and five modules of the GeoMx assay including 56 immune (GeoMx immune cell profiling panel, GeoMx IO drug target module, GeoMx immune activation status module, GeoMx immune cell typing module, GeoMx pan‐tumor module, and GeoMx myeloid module) markers was assessed by DSP. Detailed information about 56 immune markers assessed by DSP is included in Table S4.

For each slide, three or six ROIs‐located within the tumor regions annotated in the H&E slides‐ were selected. Specifically, ROIs were selected in exclusively PanCk^+^ tumoral areas; exclusively CD45^+^ immune infiltrated areas, and in combined tumoral and immune infiltrated areas (PanCk ^+^ and CD45^+^ regions). Each ROI was UV‐illuminated twice, once for the PanCk segment and once for the CD45 segment. Four or eight areas of illumination (AOIs) were collected per slide, depending on the ROI number profiled: 4 AOIs (2 PanCK^+^ tumor AOIs and 2 CD45^+^ stromal AOIs) for the samples annotated with 3 ROIs and 8 AOIs (4 PanCK^+^ tumor AOIs and 4 CD45^+^ stromal AOIs) for the samples annotated with 6 ROIs. Photocleaved oligonucleotides from each spatially resolved AOI were PCR amplified. PCR products were pooled and purified twice with AMPure XP beads (Beckman Coulter, Brea, CA). The quality of the final pool library was checked with Agilent TapeStation 4200 system (Agilent Technologies, Santa Clara, CA), and Qubit Flourometer 4.0 (Invitrogen, Waltham, MA), then denatured, normalized, and sequenced with the NextSeq 550 platform with paired‐end 27 cycles to generate FASTQ files. Once FASTQ files were generated, the files were converted to DCC files using the BaseSpace GeoMx NGS Pipeline v2.0.21 (Illumina, San Diego, CA) to be compatible with GeoMx DSP Control Center v2.4.2.2. Digital counts between AOIs were normalized with three IgG negative control isotypes (Ms IgG1, Ms IgG2a, and Rb IgG). Statistical comparisons between AOIs/group were performed by applying a linear mixed‐effect model (LMM) to account for multiple sampling of AOI segments per tissue. Differentially expressed proteins were identified by comparing AOIs between the two groups with a significance of ± Log2 Fold Change (FC) ≥ 0.6 and −log10 (*p*) ≥ 1.3.

### 2.6 Immunohistochemistry (IHC) Analysis

Immunohistochemical staining was performed on 5 μm‐thick sections derived from baseline and post‐treatment formalin‐fixed paraffin‐embedded tumor specimens using the Ventana BenchMark Ultra automated staining platform. The primary antibodies used include CD3 (clone MRQ‐39, Cell Marque™) and CD20 (clone L26, Ventana). Histopathological analyses were performed according to guidelines provided by the International TILs Working Group 2014.^22^ Briefly, the inflammatory cells were measured using visual estimation of the average percentage of positive cell staining within chosen representative tumor or stroma areas. Using visual estimation, the percentages of CD3^+^ and CD20^+^ cells were evaluated within representative areas of the invasive tumor and adjoining stroma. Of note, CD3^+^ and CD20^+^ cells found outside of the tumor border and around normal lobules were excluded. Additionally, CD3^+^ and CD20^+^ cells found in tumor zones with crush artifacts, necrosis, or regressive hyalinization were also excluded. For stromal assessments, the denominator used to determine the % stromal immune infiltration was the area of stromal tissue (i.e., area occupied by mononuclear inflammatory cells over total intertumoral stromal area), not the number of stromal cells. Comparisons of IHC results were performed using two‐tailed Mann Whitney tests or two‐tailed unpaired T‐tests, according to data distribution, with GraphPad Prism version 8.3.0 (GraphPad Software Inc., San Diego, CA).

## 3 RESULTS

### 3.1 At baseline, higher circulating lymphocytes and reduced neutrophils counts are detected in MIBC patients responding to cisplatin‐based NAC

**FIGURE 1:**
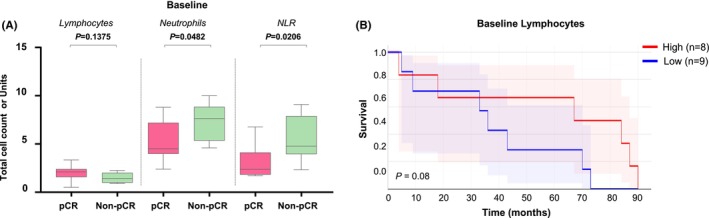
Impact of NAC cisplatin‐based therapy on circulating immune cells of patients with MIBC. *N* = 17 patients are here considered. (A) Association between lymphocytes counts, neutrophil counts, NLR and response to NAC evaluated at baseline; *p* values derived from two‐tailed Student's *t*‐tests or two‐tailed Mann–Whitney t tests, according to data distribution. (B) Association between patients' survival and levels of lymphocytes counts evaluated at baseline. Median counts set up as cut off for determining high and low.

Multiple evidences suggest that the analysis of peripheral blood immune repertoire can mirror the immune status of the TME.^23^ Consequently, we first assessed the lymphocyte counts, the neutrophil counts, and the neutrophil to lymphocyte ratio (NLR) of MIBC patients undergoing NAC. At baseline, pCR patients showed a trend of higher levels of lymphocytes and significantly lower levels of neutrophils and NLR when compared to non‐pCR patients (*p* = 0.138, *p* = 0.05, and *p* = 0.02, respectively. Figure 1A). The observations derived from our small single‐center MIBC patients' cohort are consistent with findings from previous multicenter studies that reported the association between high pretreatment NLR levels and decreased response to NAC and worse survival outcomes in MIBC patients treated with neoadjuvant chemotherapy.^24^, ^25^ Interestingly, a trend of higher levels of lymphocytes at baseline and longer OS was also observed (*p* = 0.08, Figure 1B) in our patients' cohort.

### 3.2 At baseline, higher B and T cell infiltration is detected in the TME of MIBC patients responding to cisplatin‐based NAC

**FIGURE 2:**
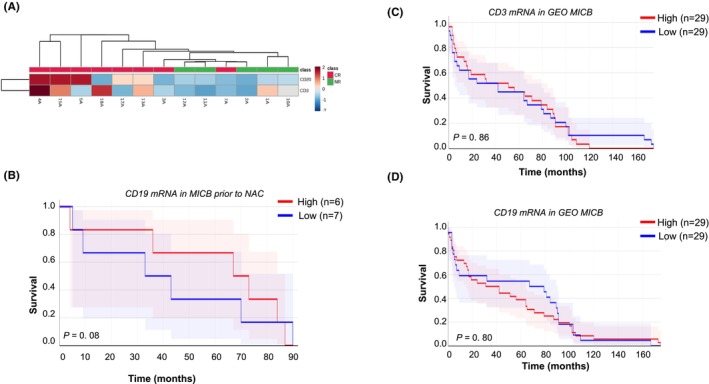
Pre‐treatment Immune Profile of MIBC specimens with diverging response to NAC‐cisplatin‐based treatment evaluated by genomic profile. (A) Supervised cluster‐based pretreatment gene expression of *CD3* and *CD20* in MIBC patients enrolled at SJCI and showing diverging response to NAC. (B) Association between B cell infiltration (evaluated based on *CD19* gene expression, median cut) and overall survival in patients of SJCI treated with NAC. (C) Association between CD3+ cell infiltration (evaluated based on *CD3* gene expression, median cut) and overall survival in MIBC patients of GEO. (D) Association between B cell infiltration (evaluated based on CD19 gene expression, median cut) and overall survival in MIBC patients of GEO.

To investigate differences present in the TME of MIBC patients characterized by diverging clinical responses to NAC treatment, we conducted whole gene expression analysis of the baseline FFPE specimens derived from 8 pCR patients and 5 non‐pCR patients. Analysis of differentially expressed genes (DEG) at baseline between the two cohorts of patients revealed the presence of 1119 transcripts (*p* ≤ 0.01; FC ≥1.5) differentially expressed between the two groups of patients. Among them, 464 genes were up‐regulated, and 655 genes were down‐regulated in pCR versus. non‐pCR, respectively (Table S1). Further evaluations conducted on the upregulated DEG in pCR versus non‐pCR revealed genes involved in B cell function (i.e., *CD19*, *MS4A1*, *CLEC4D*, *FCER2*, and *CXCR5*) and CD3 cells (*CD3G*), to be enriched in the TME of pCR patients (Figure 2A). Interestingly, further assessments also highlighted a trend of association between high baseline *CD19* mRNA and longer patients' OS following treatment (Figure 2B). Furthermore, immune‐related genes associated with inflammatory function and leukocytes trafficking such as *CCL5*, *CCL19*, *CX3CR1* were also found upregulated in the TME of pCR versus non‐pCR patients (Table S1). Functional IPA pathway analysis confirmed genes upregulated in pCR to be involved in inflammatory pathways (Table S2). Conversely, an increased expression of genes involved in cell‐to‐cell adhesion (i.e., *CDH2*, *CLDN19*, *LAMA3*, and *NRCAM*) (Table S3), DNA repair genes (i.e., *DMC1*, *HFM1*, and *RAD54B*), as well as neuronal associated genes^8^ (i.e., *GNG4*, *PEG10*, *APLP1*, and *TUBB2B*) (Table S1) was observed at baseline in the TME of non‐pCR versus pCR patients. The DNA repair genes *ATM*, *RB1*, and *FANCC*, whose DNA alterations were reported to be associated with cisplatin‐based NAC outcome,^4^ did not show statistically significant differences between pCR versus non‐pCR patients (*data not shown*). As the next step, to validate whether the elevated *CD3* and *CD19* mRNA expression levels prior to NAC chemotherapy treatment were associated with the prognosis of patients with MIBC, we performed an *in‐silico* analysis using a cohort of 58 not treated MIBC patients, available in GEO. By doing so, we observed that neither the expression of *CD3* nor *CD20* mRNA was associated with a clinical benefit in MIBC patients (Figure 2C,D), suggesting a limited role played by B cells and CD3^+^ T cells in the prognosis of MIBC patients.

### 3.3 Patterns of T and B cell infiltration in patients with MIBC undergoing NAC

**FIGURE 3:**
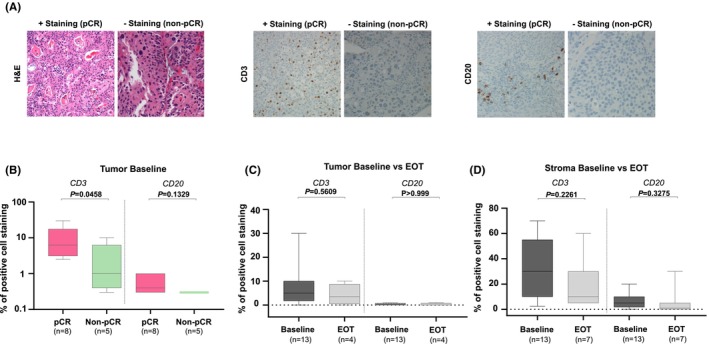
Immunohistochemistry evaluations based on baseline expression of CD3, CD20 in patients with MIBC treated with NAC. (A) Representative images of CD3 and CD20 staining in tumor baseline samples are here shown with 250X magnification. (B) Expression of CD3 and CD20 in tumor bed prior to NAC initiation. Pharmacodynamic variations of CD3^+^ and CD20^+^ cells in patients with MIBC receiving NAC evaluated in the tumor (C) or in the stroma (D). Two‐tailed Mann Whitney or two‐tailed unpaired T‐tests were used to calculate p values.

**FIGURE 4:**
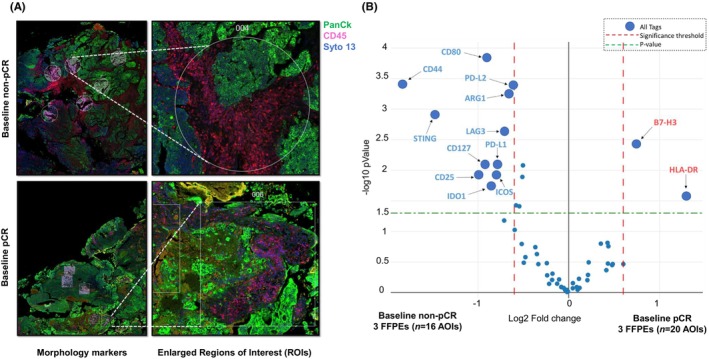
Digital Spatial profile of patients with MIBC undergoing NAC. (A) Graphic representation of Digital Spatial Profile evaluating 56 immune proteins (Table S4) in baseline FFPEs derived from non‐pCR and pCR patients. Morphology markers: PanCK (green), CD45 (pink) and DNA (blue). (B) Volcano plot highlighting immune proteins differentially expressed (FC >1.5, *p* value<0.05) at baseline between pCR (*n* = 3) and non‐pCR (*n* = 3) specimens.

To confirm the results obtained by gene expression profile, the infiltration of T cells and B cells was assessed in both the stromal areas and intratumorally with the epithelial tumor nests (Figure 3A) by using IHC. The results highlighted that, prior to NAC initiation, a significant enrichment of CD3^+^ tumor‐infiltrating lymphocytes (TILs), evaluated intratumorally, was observed in pCR patient's versus non‐pCR. A trend of increased enrichment of B cells (CD20^+^) was also observed in the same cohort of patients thus confirming the results obtained by genomic evaluation (Figure 3B). There was no difference in T and B cell infiltration within the stroma of patients with different outcomes thus demonstrating that intertumoral immune infiltration, as opposed to stromal one, correlated with response to NAC in patients with MIBC (*data not shown*). Of interest, no pharmacodynamic modulation in CD3^+^ T cells and B cell infiltrations was observed within the TME during NAC treatment (Figure 3C,D). To further explore differences in immune‐ biology occurring in the TME of patients with different responses to NAC, a subset of six FFPE specimens derived from three pCR and three non‐pCR patients, respectively, were subjected to protein digital spatial profiling (DSP) based on a panel of 56 immune‐related markers using Nanostring GeoMx. The Digital Spatial Profiling results revealed a high immunosuppressive and immune exhausted niche to be present in the TME of non‐pCR compared with pCR patients prior to cisplatin‐based NAC. In particular, the DSP results highlighted an increased infiltration of Tregs (CD25^+^, CD127^+^, IDO1^+^) and immunosuppressive cells (ARG^+^) at baseline in non‐pCR versus pCR patients. Interestingly, also an increased expression of immune checkpoints such as PD‐L1, ICOS, STING, and LAG3, was observed at baseline in the TME of non‐pCR versus pCR. Conversely, an increased expression of HLA‐DR^+^ cells and the immunoregulatory protein B7‐H3 (CD276) was observed in the TME of pCR prior to treatment initiation (Figure 4A,B; Table S4).

## 4 DISCUSSION

Platinum‐based chemotherapy combinations have proven effective in treating patients with advanced urothelial carcinoma,^26^ and several studies have explored the impact of neoadjuvant chemotherapy (NAC) and adjuvant chemotherapy (AC) in MIBC.^27^ The main goals of NAC include the reduction of the primary tumor size to enhance the chance of complete surgical resection (R0), and the treatment of any distant micro metastases present at diagnosis. Achievement of pCR after NAC has been associated with improved clinical outcomes including lower cancer recurrence rate and increased overall survival as compared to patients without pCR. Both NAC and AC have hypothetical benefits and disadvantages in the treatment of MIBC however clinical evidence is more robust in support of NAC based on randomized clinical trials.^3^ With NAC, it is possible to objectively evaluate the effectiveness of treatment and to investigate the biological mechanisms underlying resistance to NAC through the genetic analysis of tumor tissue collected before and after treatment. However, not all patients will respond to NAC, and delaying cystectomy in such cases could have a deleterious effect. Hence, identifying features associated with response to NAC treatment in MIBC can allow a better patient selection strategy offering alternative treatments in patients not likely to respond thus avoiding unnecessary toxicities and delay in cystectomy. In recent years, advancements in the molecular biology of bladder cancer through DNA sequencing and transcriptomic analysis of mRNA, have enabled the identification of potential predictive biomarkers of response to cisplatin‐based chemotherapy. Furthermore, it has become possible to establish subtypes of bladder cancer based on gene expression profiles that correlate with specific patient's clinicopathological characteristics and with response to anti‐tumor treatments. In this study, we evaluated clinical‐immune‐based assessments in the peripheral blood, and we applied complementary molecular and proteomic methodologies to analyze the TIME in MIBC patients undergoing neoadjuvant chemotherapy with the objective to identify biomarkers associated with NAC clinical benefit and features that could pave the way for innovative treatment options. To evaluate differences in the peripheral immunologic characteristics among MIBC patients treated with cisplatin‐based NAC, we assessed differences of absolute lymphocyte and neutrophil counts, as well as NLR levels prior NAC treatment. Interestingly, pCR patients showed a trend of higher levels of lymphocytes and significantly lower levels of neutrophils and NLR, when compared to non‐pCR patients at baseline, findings consistent with previous large multicenter studies which highlighted the predictive and prognostic role of the NLR ratio in MIBC patients treated with neoadjuvant chemotherapy.^24^, ^25^

In this study, molecular assessments performed on FFPEs prior to NAC treatment revealed a higher infiltration of CD3^+^ T cells and B cells in pCR versus non‐pCR patients. Interestingly, a trend of association between high baseline *CD19* mRNA and longer patients' OS following treatment was also found, suggesting the predictive value of infiltrated B cells in the TME of MIBC patients prior to NAC therapy. Of note, the results derived from the Digital Spatial Profiling also highlighted that patients not likely to respond to NAC are characterized at baseline by an immune compromised microenvironment with increased infiltration of immunosuppressive cells (i.e ARG1^+^ cells and T regs) and cells expressing ICOS, LAG3 and STING checkpoints. These findings suggest that, in MIBC setting, the existence of a functional immunity prior to NAC initiation will lead to an ongoing immune response that may be able to suppress tumor progression.

Controversial results have been obtained when evaluating the relationship between the pretreatment TIME and clinical benefit of NAC, emphasizing the necessity for a deeper understanding of the pretherapy TME immune architecture of MIBC patients. Ikarashi et al^28^ showed that preexisting CD8^+^ TILs and CD204^+^ cells (M2 macrophages) in baseline tissues were associated with a poorer NAC prognosis in MIBC patients. On the contrary, a recent study in metastatic urothelial carcinoma suggested that cisplatin‐based therapy only improves upon adaptive immunity that already is present before in the TME before the patients receive treatment.^29^ Another NAC study conducted by van Wilpe et al,^30^ showed a difference in TIL density as well in patients with or without recurrence. Patients who had decreased TIL density had early recurrence, whereas patients with higher density of CD3^+^ and CD3^+^CD8^+^ TIL had less cancer recurrence even in the tumor tissue prior to cystectomy.^30^ Taken together these findings support a role of cisplatin‐based regimens in antitumor immunomodulation thus highlighting that the anticancer activity of cisplatin‐based regimens is not limited to the ability to inhibit mitosis and to act of DNA repair machinery.

To add, additional studies also reported a relationship between MIBC patient molecular stratification and response to cisplatin‐based NAC. However, there are conflicting results concerning using molecular subtyping for MIBC treatment decision as well. While Taber et al^9^ showed lower response rate for patients with basal tumors, Seiler at al^10^ observed increased overall survival after NAC treatment in patients with basal tumors. Sjödahl et al^11^ reported patients with luminal tumors had the highest response to cisplatin‐based NAC. However, Lotan et al^12^ revealed patients with luminal tumors experienced a minimal survival benefit from NAC treatment. These conflicting results suggest that consideration of additional and simpler patients' selection strategies are necessary to elucidate the mechanisms underlying NAC response. Furthermore, the application of comprehensive molecular profiling in clinical practice ongoing in community hospitals is challenging due to cost constraints and limited number of patients. For all these reasons, in this exploratory study we suggest that patient's baseline intra‐tumoral immune stratification might be an easiest and simplest method to help guide clinical benefit from cisplatin‐based NAC.

Therefore, it is instrumental to monitor in prospective clinical trials various components of the immune system (and their response to cisplatin‐based regimens) to reach new levels of confidence on the impact of the immune environment in patients' stratification and response to NAC. The field of bladder cancer is evolving rapidly with checkpoint inhibitors (CPI) and antibody drug conjugates (ADC) playing increasingly important role in the treatment of advanced disease.^31^, ^32^ It is very probable that these compounds will become useful in the perioperative management of urothelial carcinoma and multiple ongoing clinical trials are investigating that concept.^33^ However, it is highly unlikely that cisplatin‐based chemotherapy will be completely replaced in this setting based on robust complete responses it elicits in substantial fraction of patients. Currently available CPI and ADC regimens have their own limitations and side effects and may not be applicable and beneficial to all patients. One can envision future with progressively nuanced application of various drugs in the perioperative setting based on histological variants, tumor genomic, immune, molecular factors, and clinical patient characteristics. Appropriate individualized selection of increasingly diverse treatment options will become even more important as the field of therapy of bladder cancer continues to evolve.

Limitations of our study include the small number of patients studied. Therefore, one should interpret the results with caution, particularly the survival analysis. Given the exploratory nature of this study, further research is underway to validate our findings by setting up novel biomarker based clinical studies in MIBC. In doing so, we do consider that the expansion of intra tumoral immunity may be a useful strategy for the development of more effective treatments' programs in patients with MIBC undergoing NAC.

## 5 CONCLUSION

The findings of this study might suggest that the general approach used to identify biomarkers predicting the clinical outcome of MIBC patients treated with NAC should be reevaluated thus considering also the impact played by the immune environment in treatment response.

## AUTHOR CONTRIBUTIONS

**Alberto Mendoza‐Valderrey:** Data curation (equal); formal analysis (equal); investigation (equal); methodology (equal); project administration (equal); writing – original draft (equal); writing – review and editing (equal). **Jane Choe:** Data curation (equal); project administration (equal); writing – review and editing (equal). **Daria M. Kessler:** Formal analysis (equal); methodology (equal); writing – review and editing (equal). **Gianna Jimenez:** Project administration (equal); writing – review and editing (equal). **Xinmin Li:** Methodology (equal); writing – review and editing (equal). **Steven Kolker:** Writing – review and editing (equal). **Warren Allen:** Methodology (equal); writing – review and editing (equal). **Jennifer A. Linehan:** Writing – review and editing (equal). **Przemyslaw W. Twardowski:** Data curation (equal); writing – review and editing (equal). **Maria Libera Ascierto:** Conceptualization (lead); data curation (lead); formal analysis (lead); investigation (lead); methodology (lead); supervision (lead); writing – original draft (lead); writing – review and editing (lead).

## FUNDING INFORMATION

This study was supported by a generous donation received from the Rosalie and Harold Rae Brown Foundation.

## CONFLICT OF INTEREST STATEMENT

J.A.L. is a speaker and consultant for Urogen, Intuitive Surgical, and Medtronic. All other authors declare no conflicts of interest related to this project.

## ETHICS STATEMENT

The study was conducted in accordance with the Declaration of Helsinki and approved by the Joint Institutional Review Board (or Ethics Committee) of SJHC/SJCI (formerly John Wayne Cancer Institute): SJCI Universal Consent (Providence Health System Portland IRB: SJCI/JWCI‐18‐0401) and Western IRB: MORD‐RTPCR‐0995. All specimens evaluated in this study were derived from consenting patients.

## Supporting information


**Table S1.** List of Illumina probes differentially expressed (*p* ≤ 0.01; FC ≥1.5) in pCR versus non‐pCR MIBC patients prior to neoadjuvant chemotherapy.

**Table S2.** IPA enrichment analysis of genes involved in Inflammatory pathways are found enriched in pCR versus non‐pCR MIBC patients undergoing neoadjuvant chemotherapy.

**Table S3.** IPA enrichment analysis of genes involved in cell–cell adhesion is found enriched in non‐pCR versus pCR MIBC patients undergoing neoadjuvant chemotherapy.

**Table S4.** List of the 56 selected immune‐related proteins assessed by DSP in pCR versus non‐pCR MIBC patients prior to NAC treatment.

## Data Availability

Gene expression data discussed in this study have been included in NCBI's Gene Expression Omnibus^34^ and are accessible through GEO Series accession number GSE247185.
